# Biosorption of methyl blue onto tartaric acid modified wheat bran from aqueous solution

**DOI:** 10.1186/1735-2746-9-16

**Published:** 2012-12-05

**Authors:** Shuhua Yao, Hong Lai, Zhongliang Shi

**Affiliations:** 1 School of Applied Chemistry, Shenyang University of Chemical Technology, Shenyang, China

**Keywords:** Modified wheat bran, Biosorption, Methyl blue

## Abstract

Tartaric acid modified wheat bran was utilized as adsorbent to remove methyl blue, a basic dye from aqueous solution. Batch experiments were carried out to study the effect of various experimental parameters such as initial solution pH, contact time, initial dye concentration and adsorbent dosage, on dye adsorption. The results showed that the modification of wheat bran by tartaric acid significantly improved its adsorption capacity, and made this material a suitable adsorbent to remove methyl blue. The adsorption capacity of modified wheat bran was about 1.6 times higher than that of unmodified one. The amount of methyl blue adsorbed was found to vary with initial solution pH, adsorbent dosage, contact time and initial methyl blue concentration. Kinetics study showed that the overall adsorption rate of methyl blue was illustrated by pseudo-second-order kinetic model. The applicability of the Langmuir and Freundlich models for the data was tested. Both models adequately described the experimental data of the biosorption of methyl blue. The maximum adsorption capacity for methyl blue calculated from Langmuir model was 25.18 mg/g. The study has shown the effectiveness of modified wheat bran in the removal of methyl blue, and that it can be considered as an attractive alternative to the more expensive technologies used in wastewater treatment.

## Introduction

Improper dye discharge from various industries such as textile, paper, cosmetic and plastics into receiving streams can be one of the sources of water pollution. The release of these effluents not only causes various disruptions in the ecosystems, but also poses hazard effects as most of the dyes are highly toxic, mutagenic and carcinogenic in nature. Therefore, discharge regulations are progressively becoming more stringent.

Chemical oxidation, chemical coagulation, biological treatment and photodegradation are some of the most commonly practiced processes for the removal of dyes from industrial wastewaters [1-3]. However, these methods are non-economical and have many disadvantages such as high reagent and energy requirements, generation of toxic sludge or other waste products that require disposal or treatment. In addition, various dyes used in the industry are particularly difficult to remove by conventional waste methods as they are stable to light and oxidizing agents and resistant to aerobic digestion [4]. There is thus a need to search for new processes that could remove dyes commonly used in the industry. The adsorption technique is one of the preferred methods for removal of dyes because of its efficiency and low cost.

Activated carbon is the most popular and widely used adsorbent but it is expensive and its cost increases with the quality. In addition its regeneration with refractory technique results in a 10-15% loss of the sorbent and its uptake capacity. Thus, there has been intensive research exploring the potential of alternative low-cost materials as sorbents for dyes. For this purpose in recent years, various biological and industrial by-products have been investigated intensively for their ability to remove dye from aqueous solution, as they can be obtained readily and are in great abundance, such as corncob [5], chitosan [6], waste coir pith [7], giant duckweed [8], powdered peanut hull [9], coccinea berries [10], trees’ leaves [11], eggshell [12], modified clays [13], fly ash [14], beer brewery waste [15], natural zeolite [16], bentonite [17], rice bran [18] and rice hull [19]. Among these materials, some biosorbents have shown extraordinary properties for dye removal.

The bran of wheat is the shell of the wheat seed and contains many nutrients of wheat. This bran is usually removed in the processing of wheat into flour. It is environmentally friendly and is nutritious to the plants. Therefore the use of wheat bran to eliminate pollution from water and wastewater is interesting. There are a few reports of heavy metal adsorption by wheat bran, as a by-product of a flour factory, such as Cr(VI) [20], Pb(II) [21], Cu(II) and Cd(II) [22]. The purpose of the present study was to *a*) investigate the adsorption of the cationic dye methylene blue (MB) on to wheat bran modified with tartaric acid bran; *b*) study the effect of different parameters such as contact time, initial pH, adsorbent dosage and initial MB concentration on adsorption process and; *c*) find optimum adsorption isotherm as well as the rate of adsorption kinetics.

## Materials and methods

### Preparation of adsorbent

The wheat bran used in this study was a by-product of a flour factory in Shenyang, China. The wheat bran was washed thoroughly with water to ensure the removal of dust and ash. It was then sieved to ~50 mesh size by passing the milled material through standard steel sieves to remove any large non-wheat bran solids. Then, about 4.0 g grinded wheat bran was mixed with 30 mL of 1.2 mol/L tartaric acid. The mixture was stirred until homogenous and dried at 50°C for 24 h. The modified wheat bran was subsequently washed with distilled water until neutral and dried at 50°C for 24 h.

### Preparation of methlylene blue solution

Methlylene Blue (C_16_H_18_N_3_SCl·3H_2_O, a cationic dye) was purchased from Sanaisi reagent Ltd. (Shanghai, China) and used without further purification. The wavelength of maximum absorption (λ_max_) of this dye is 664 nm. Methlylene Blue was dried at 110°C for 2 h before use. All of the methlylene blue solution was prepared with distilled water. The stock solution of 1000 mg/L was prepared by dissolving methlylene blue in 1000 mL distilled water. The experimental solution was obtained by diluting the stock solution in accurate proportions to different initial concentrations with distilled water.

### Batch adsorption experiments

Batch techniques were used to investigate MB adsorption, which was examined via kinetic studies and adsorption isotherms, together with the effect of some operating parameters. All batch experiments were carried out in duplicate and the results given are the means with a relative standard deviation of less than 5%. Control experiments without sorbent were carried out to ascertain that the sorption was by the adsorbent and not the wall of the container.

The comparison study on the uptake of MB by unmodified and modified wheat bran was carried out at room temperature (20±1°C) by mixing 0.5 g of adsorbent with 100.0 mL of MB solution (100 mg/L) in a glass vial at pH=7.0, and shaken at 150 r/min for 1 h.

The adsorption kinetic study was performed for MB in solution at pH=7.0 and room temperature (20±1°C). Several glass vials were used to hold 100mL MB solution of known initial concentration (50, 100, and 200 mg/L) and 0.5 g of adsorbent at pH=7.0, and shaken at 150 r/min for a duration ranging from 0 to 360 min. At certain period of time, each vial was removed from the shaker, and the solution was then centrifuged at 3000 r/min to measure the MB concentration. In order to investigate the mechanism of MB adsorption on the modified wheat bran, the pseudo-second-order rate equation model was applied to the kinetic experimental data. The pseudo-second-order kinetic equation could be derived as [23]:

(1) $$\;t/q_{t}=1/\left(k_{2}q_{e}^{2}\right)+t/q_{e}$$

The adsorption capacity of modified wheat bran was determined by batch adsorption isotherms at 20°C in aqueous solution (initial pH values fixed at 3, 5 and 7). In several glass vials, 100 mL of solution containing various MB concentrations (50, 100, 150, 200, 250 mg/L) were contacted with 0.5 g of modified wheat bran. The vials were placed in a water bath at 20°C and shaken at 150 r/min for approximately 8 h, and the pH was adjusted by adding 0.1 mol/L NaOH or HNO_3_ (±0.10). The reaction mixture was then centrifuged at 3000 r/min, the MB concentrations of the various filtered solutions were analyzed by measuring the absorbance at the maximum wavelength of 664 nm using a spectrophotometer (VIS-7220, Beijing, China). The amount of MB adsorbed was calculated from the following equation:

(2) $$\;q_{e}=V\left(C_{0}-C_{e}\right)/W$$

where *q*_e_ is the amount of MB sorbet onto 1.0 g of the adsorbent (mg/g), *C*_0_ and *C*_e_ are the initial and equilibrium MB concentrations in the solution (mg/L), respectively; V is the solution volume (L); and W is the mass of modified wheat bran (g).

The results of MB adsorption on modified wheat bran were analyzed by using Langmuir and Freundlich isotherm models to evaluate parameters associated to the adsorption behavior. The linear form of Langmuir equation at a given temperature is represented by:

(3) $$\;c_{e}/q_{e}=1/\left(b\times q_{m}\right)+c_{e}/q_{m}$$

The linear form of Freundlich model can be expressed by the following equation:

(4) $$\;lgq_{e}=lgk_{f}+\left(1/n\right)lgc_{e}$$

Where b is the adsorption constant (L/mg) related to the energy of adsorption and represents the affinity between the adsorbent and adsorbate, *q*_m_ is the maximum adsorption capacity (mg/g), *k*_f_ and n are constants related to the adsorption capacity and affinity, respectively.

To determine the effects of different parameters on MB adsorption, experiments were performed at various initial pH values ranging between 3 and 10. Initial concentrations of 100 mg/L MB and 0.5 g adsorbent per 100 mL of solution were employed. The suspensions were shaked at 150 r/min for 1 h. Then optimum initial pH was identified. The effects of adsorbent dosage, initial solution pH, initial dye concentration and contact time were conducted.

## Results

**Figure 1:**
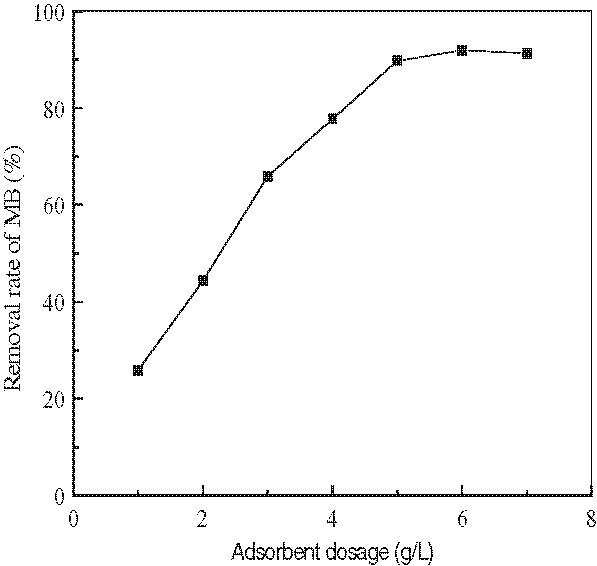
**Effect of adsorbent dosage on the adsorption of MB.** (Experiment conditions employed: initial MB concentration 100 mg/L, solution pH 7.0, adsorption time 1 h, agitation speed =150 r/min).

**Figure 2:**
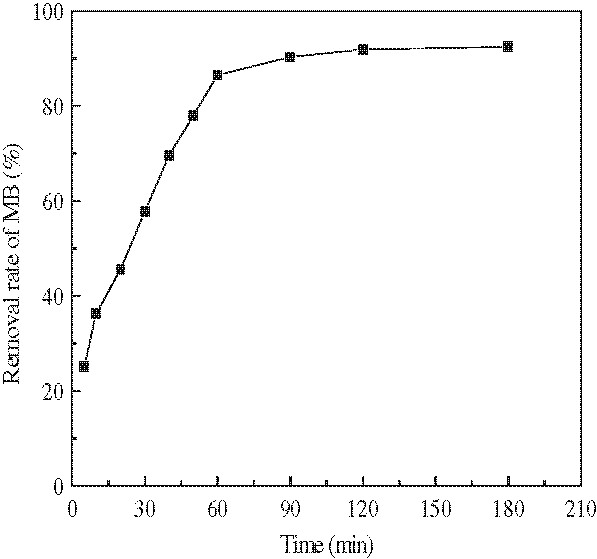
**Effect of contact time on the adsorption of MB.** (Experiment conditions employed: initial MB concentration 100 mg/L, adsorbent dosage 5.0 g/L, solution pH 7.0, agitation speed =150 r/min).

**Figure 3:**
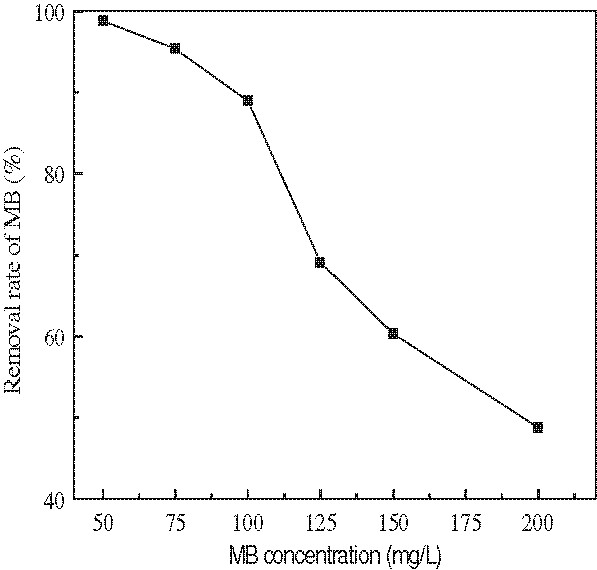
Effect of initial MB concentration on the adsorption of MB.(Experiment conditions employed: adsorbent dosage 5.0 g/L, solution pH 7.0, adsorption time 1 h, agitation speed =150 r/min).

**Figure 4:**
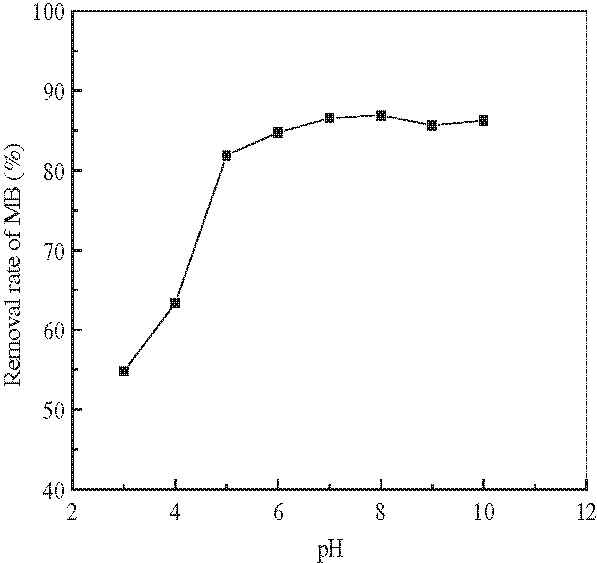
Effect of pH on the adsorption of MB.(Experiment conditions employed: initial MB concentration 100 mg/L, adsorbent dosage 5.0 g/L, adsorption time 1 h, agitation speed =150 r/min).

Comparison of the results on the uptake of MB by unmodified and modified wheat bran showed that the removal rates of MB was 55.32% and 85.76%, respectively. As presented in Figure 1, the effect of changing adsorbent dosage on adsorption of concentration of 100 mg/L of MB can be seen. Figure 2 demonstrates the effect of contact time on adsorption of concentrations of 100 mg/L of MB by a fixed dosage of modified wheat bran. Figure 3 shows the effect of changing initial MB concentrations (50, 75, 100, 150 and 200 mg/L) on adsorption of MB by a fixed constant temperature (20°C), pH=7.0, contact time (1 h) and adsorbent dosage (5.0 g/L). Effect of initial solution pH on adsorption process of MB at a constant contact time of 1 h can be seen in Figure 4.

**Figure 5:**
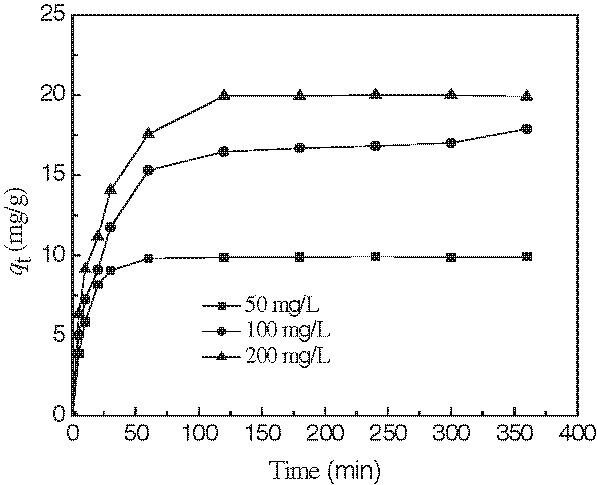
Adsorption kinetics of MB by modified wheat bran.(Experimental conditions employed: pH = 7.0, agitation speed =150 r/min, adsorbent dosage = 5.0 g/L).

**Figure 6:**
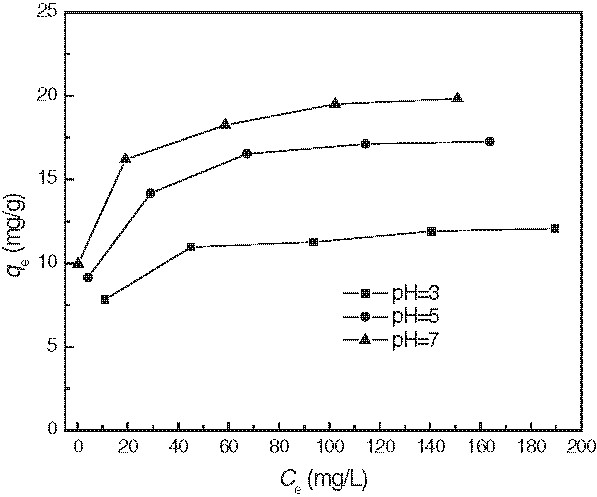
Adsorption isotherms for MB by modified wheat bran.(Experimental conditions employed: adsorbent dosage = 5.0 g/L, adsorption time 8 h, agitation speed =150 r/min).

In Figure 5, the results of the change of MB concentration with adsorption time for an initial concentration of 50, 100, 200 mg/L and pH=7.0 and adsorbent dosage of 5.0 g/L were presented. Table 1 demonstrates the results of kinetic parameters for MB adsorption by modified wheat bran. The results of developing adsorption isotherm for MB adsorption by modified wheat bran are presented in Figure 6. The isotherms showed that the adsorption capacity increased with increasing equilibrium concentration of MB. Table 2 demonstrates the results of determining the parameters of Langmuir and Freudlich equations for MB adsorption by modified wheat bran.

**Table 1:** Kinetic parameters for MB Adsorption by modified wheat bran

***C***_**0 **_**(mg·L**^**-1**^**)**	***q***_**e**_**(mg·g**^**-1**^**)**	***k***_**2**_**(L·mg**^**-1**^**.min**^**-1**^**)**	**R**^**2**^
50	10.11	2.15×10^−2^	0.9997
100	18.28	3.47×10^−3^	0.9995
200	20.83	2.03×10^−3^	0.9993

**Table 2:** The parameters of Langmuir and Freudlich equation

**Initial pH**	**Langmuir equation**			**Freundlich equation**		
	***q***_**m**_**(mg·g**^**-1**^**)**	**b(L·mg**^**-1**^**)**	**R**^**2**^	**1/n**	**K**_**f**_	**R**^**2**^
3.0	16.46	0.1811	0.9997	0.1810	5.6865	0.9605
5.0	21.83	0.3175	0.9998	0.1508	7.3164	0.9811
7.0	25.18	0.6636	0.9996	0.1099	11.6262	0.9991

## Discussion

Results on the uptake of MB showed that the adsorption of MB was enhanced when modified wheat bran was used as the adsorbent. The adsorption capacity of modified wheat bran for MB is about 1.6 times higher than that of unmodified wheat bran, and it has higher removal efficiency for basic dye than some other biosorbents [19,24]. The presence of carboxyl groups in modified wheat bran is believed to be primarily responsible for the adsorption of MB. Previous investigations have also postulated that the adsorption of positively charged species is due to the presence of binding sites such as carboxyl and hydroxyl groups on the surface [19,25,26].

It can be seen from Figure 1 that the removal efficiency of MB considerably increased with the increase of adsorbent dosage. This is because of the availability of more and more adsorption sites (carboxyl groups) for MB adsorption during the adsorption reaction. A further increase in adsorbent dosage (>5.0 g/L) did not cause significant improvement in MB adsorption. This may be due to the adsorption of almost all MB to the adsorbent and the establishment of equilibrium. As shown in Figure 2, the adsorption was very fast and equilibrium between the aqueous solution and modified wheat bran was established within about 1 h. Then, there was no significant change in MB removal rates by further increasing the time. Similar results have been reported in literature for removal of dyes [27,28].

It can be seen from Figure 3 that the MB removal rates decreased with the increase in initial MB concentration, the percentage adsorption of MB on modified wheat bran decreased from 98.5 to 43.6% as the initial MB concentration was increased from 50 to 200 mg/L. At lower MB concentrations, the ratio of the available adsorption sites of the adsorbent to the initial number of molecules of MB is large and subsequently the fractional adsorption becomes independent of initial concentration. However, at higher concentrations, the available sites of adsorption become fewer, and hence the percentage removal of MB which depends upon the initial concentration, decreases.

It is evident from Figure 4 that the percentage of MB removal strongly depended on the solution pH. The removal of MB increasesd dramatically with increasing pH at pH=3~5, and when the solution pH was 6~10, the increases slowed with increasing pH. At low pH, the removal of MB was suppressed by H^+^ ions that surrounded the surface of the adsorbent hindering the approach of MB to the carboxylate groups present on the surface of modified wheat bran. The protonation of carboxylate groups would also reduce the MB adsorption. With increasing pH, adsorption of MB became favorable due to the deprotonation of carboxyl groups, resulting in more adsorption sites available for binding with MB. This phenomenon favors the adsorption of positively charged dye due to electrostatic attraction [7].

The kinetic experimental data of MB on the modified wheat bran (Figure 5) was simulated by pseudo-second order rate equation (1). From the results listed in Table 1, it can be seen that the kinetic data could be described well by the pseudo-second order rate equation which was based on the assumption that the rate limiting step may be the chemical sorption or chemisorptions involving valency forces through sharing or exchange of electron between adsorbent and adsorbate [29]. This result was accordant with previous report [19]. However, Hu *et al*. showed that MB adsorbed on other biosorbents follows Lagergreen’s pseudo-first order rate equation [30].

Experimental isotherm data acquired at different initial pH (Figure 6) were correlated with the linear form of Langmuir model. From the isotherm parameters related to the model (Table 2), it could be seen that both q_m_ and b increased with increasing initial pH from 3.0 to 7.0. The maximum adsorption capacities (q_m_) were 16.483, 21.864 and 25.092 mg/g at pH of 3, 5 and 7, respectively. Compared with other biosorbents [31], the modified wheat bran has a much bigger adsorption capacity for MB. High values of b were reflected in the steep initial slope of an adsorption isotherm, indicating desirable high affinity. Therefore, modified wheat bran performed well in MB adsorption at initial pH=7.0 compared to other initial pH values examined.

The Freundlich isotherm model was also used to analyze the results of MB adsorption on modified wheat bran (Figure 6). Experimental isotherm data acquired at different pH were fit with the linear form of Freundlich model. The isotherm parameters related to the model (Table 2) showed that the *k*_f_ constant was increased with the increase of initial pH values, at initial pH=7.0, *k*_f_ reached its corresponding maximum value, and 1/n value at initial pH=7.0 was smaller than that at other initial pH values. These implied that the affinity between the adsorbent and MB molecules was also higher than other initial pH values. From the results in Table 2, the correlation coefficients values (R^2^) were found to be in the range of 0.9605 to 0.9998 for two models; it could be concluded that the adsorption isotherm of MB on modified wheat bran followed the Langmuir and Freundlich models. Applicability of both isotherms to adsorption of dyes by treated spent bleaching earth, activated carbons and agricultural wastes have been reported previously [19,32-34].

## Conclusion

The modification of wheat bran by tartaric acid significantly improved its adsorption capacity due to its concentration of carboxylic groups, and made this material a suitable adsorbent to remove MB, from aqueous solutions. The adsorption capacity of modified wheat bran for MB was about 1.6 times higher than that of unmodified wheat bran. The amount of MB adsorbed was found to vary with initial solution pH, adsorbent dosage, contact time and initial MB concentration. The overall adsorption rate was illustrated by the pseudo-second-order kinetic model. The equilibrium data obtained from this study was well presented by Langmuir model. As wheat bran is readily available in great abundance in China, it can be considered as an attractive alternative to the more expensive technologies used in wastewater treatment.

## Competing interests

The authors declare that they have no competing interests.

## Authors' contributions

YSH, LH and SZL 1) have made substantial contributions to conception and design, or acquisition of data, or analysis and interpretation of data; 2) have been involved in drafting the manuscript or revising it critically; and 3) have given final approval of the version to be published. All authors read and approved the final manuscript.

## Authors' information

YSH received B. Sc. in Chemistry (Northeastern Normal University, China), M. Sc. in Physical Chemistry (Northeastern Normal University, China) and Ph. D. in Environmental Science and Technology (Northeastern University, China). Currently Professor, School of Applied Chemistry, Shenyang University of Chemical Technology, China.

LH received B. Sc. in Applied Chemistry (Shenyang University of Chemical Technology, China. Currently M.S. student in Applied Chemistry, Shenyang University of Chemical Technology.

SZL received B. Sc. in Chemistry (Northeastern Normal University, China) and M. Sc. in Physical Chemistry (Northeastern Normal University, China). Currently Associate Professor, School of Applied Chemistry, Shenyang University of Chemical Technology, China.
